# Boys’ Experience of Physical Education When Their Gender Is in a Strong Minority

**DOI:** 10.3389/fpsyg.2021.573528

**Published:** 2021-03-15

**Authors:** Pål Lagestad, Eero Ropo, Tonje Bratbakk

**Affiliations:** ^1^ Faculty of Education and Arts, Nord University, Levanger, Norway; ^2^ Faculty of Education and Culture, Tampere University, Tampere, Finland

**Keywords:** physical education, high school, gender, minority boys, physical fitness

## Abstract

A literature search indicates an absence of research into boy’s experiences of physical education (PE) in classes in which there is a significant majority of girls. The aim of the study was to examine how boys in such classes experience their PE lessons. The methodological approach was qualitative, and data were collected with interviews of 13 boys in classes with more than 90% girls at a Norwegian high school. The data were analyzed with QSR NVivo 10 (London), focused on creating categories of meaning, in which students’ experiences were taken as subjectively true. The data are based on subjective constructions, which students constructed as part of their own interpretations and reflections on what had occurred in PE at the school. Results of the study came out in the form of three main findings. Two of those relate to a negative experience and the third to a positive experience of PE. The boys mostly felt that they are physically superior and have to consider the girls. Furthermore, the boys reported little challenge and feelings of mastery while being together with passive girls who are allowed to choose the activities. However, the boys found it easier to show off in front of the teachers and classmates when there were just a few boys in the class. The results are discussed in relation to gender-related theory on how the respondents are producing a traditional male gender in PE through their mastery, strength, and ambition to compete. We suggest a new approach of teaching that is more student-centered. A strategy could be to include other activities than sport-based activities into PE – activities that do not require strength and other athletic skills leading to feelings of hegemonic masculinity. A larger focus on social interactions during PE classes – activities in which students’ sex is not as important as in traditional teacher- and sport-centered PE classes, may be a good strategy.

## Introduction

Until 1970, it was normal for boys and girls in Norway to have separate physical education (PE) classes, and the introduction of curriculum reforms in 1974 led to mixed classes (Klomsten, 2013). Other Western countries, such as the United States, Canada, England, Australia, the Netherlands, Finland, and Sweden, have legislated for the introduction of mixed PE classes (Macdonald, 1990; Vertinsky, 1992; Van Essen, 2003; Annerstedt, 2008; Klomsten, 2013; Finnish National Agency for Education, 2020). This development in Norway arose as a consequence of gender equality work in both the whole society and schools, something that had been taking place in the years after the second world war. The main argument for the introduction of mixed PE classes was that all students should have equal opportunities and equal rights for participation, something that could form a basis for a fairer learning environment (Hedlund et al., 1999). Today, boys and girls have the same lesson plan for PE.

In middle school, the two genders are relatively equally represented in class because the same number of boys and girls are born in Norway (SSB, 2019). In high school, the students make choices in relation to their wishes and interests. Considering that girls and boys have differing interests in different areas of study, different proportions of girls and boys arise in many classes in high school (Bufdir, 2018), particularly in vocational training in which, for example, it is more typical that girls choose design and crafts or health and child development. In the Norwegian schools, PE contributes to the students’ experience of joy, mastery, and inspiration, and a lifelong delight in physical activity is one of the main aims (Utdanningsdirektoratet, 2015b). Drummond (2003) also highlights the PE activities as important and crucial in the development of boys’ identity. However, a study found that boys in girl-dominated classes differentiate from other students by having the highest level of absence from PE classes (Lagestad et al., 2017) and point toward the importance of more research to explain why. Taking this finding into account, the way in which boys in classes when they constitute a small minority among many girls experience joy, mastery, and inspiration in PE is an adequate question.

A literature search indicates that gender-related research in PE is most often concerned with girls’ well-being, inclusion, and engagement (Garrett, 2004; Oliver and Lalik, 2004; Oliver et al., 2009; Hill, 2015a; Oliver and Kirk, 2016; Lamb et al., 2018) even if there are studies focused upon boys’ relation to PE (Davison, 2000; Drummond, 2003; Tischler and McCaughtry, 2011, 2014; Hill, 2015b; Gerdin, 2017; Gerdin and Larsson, 2018; Monney and Gerdin, 2018). Benefits of single-sex vs. mixed sex PE has been studied in a few studies concerning, for instance, students’ activity levels or heart rate in single-sex or mixed groups (e.g., Mutz and Burrmann, 2014; Póvoas et al., 2018; Wallace et al., 2020). Also, gender studies concerning experiences of sexual minorities in sports and PE have increased in the last few years (see, e.g., Kavoura and Kokkonen, 2020). However, research into boys’ experiences of PE in classes in which girls are in a clear majority seems to be missing.

The main aim of PE in Norwegian schools is to enhance an active lifestyle and lifelong pleasure in movement (Utdanningsdirektoratet, 2019). Well-being, mastery, happiness, and activity level in PE are important indicators related to the achievement of these main aims. Norwegian PE is teacher- and sport-centered (Standal et al., 2020). Hill (2015b) argues that student status in PE contexts is often associated with performance of highly proficient sporting bodies, and certain bodily appearance and performance, such as ability, strength, or muscularity, attain high status. Research shows that students are excluded from PE because they do not have the physical skills to gain legitimacy from other students (Munk and Agergaard, 2015). Using interviews, Hill (2015b) shows how boys invested in their bodies by doing physical activities that enabled them to develop muscularity and fitness in PE. The argument that PE is experienced by the students to be closely related to sport and masculinity is supported by several studies (Davison, 2000; Drummond, 2003; Tischler and McCaughtry, 2011, 2014). Physiologically speaking, researchers have argued that boys are better placed to perform well in PE because they, in general have better physical prerequisites for developing the basic elements, such as strength, flexibility, and endurance (Evans, 1989; Lagestad, 2017). In general, boys are also taller and weigh more than girls of the same age (Lagestad, 2017) even if the group of boys never will be a homogeneous group, and girls can be taller and stronger than boys. Height, weight, endurance, strength, and flexibility are advantageous qualities to have in most of the activities, especially ball games, which form a large part of the PE (Standal et al., 2020). Evans (1989) points to, for example, boys’ physical superiority and size giving them the possibility of dominating at basketball. Evans was early in making the point that PE teachers who continually make use of “boys’ activities” contribute to the legitimization of PE as an arena for boys.

A number of theories are relevant for understanding boys’ experiences of having PE in classes in which girls form a substantial majority. Tischler and McCaughtry (2014) point out that researchers who examine the male experience in PE have used a masculinity theory as their primary framework and that the theory of hegemonic masculinity is a central concept (Connell and Messerschmidt, 2005). Hegemonic masculinity is a relational concept that is produced as the dominant concept that legitimates inequitable hierarchical relations between boys and girls. Even if a study shows that negotiation of the gender discourses and power relations may take place (Gerdin, 2017), boys in PE classes with a majority of girls may produce a traditional male gender and hegemonic masculinity in PE (Gerdin and Larsson, 2018; Monney and Gerdin, 2018; Barker, 2019).

The universal characteristics of hegemonic masculinities in PE are related to strength, speed, muscularity, athleticism, aggression, and competitiveness, and the production of those characteristics is often influenced by social forces (Tischler and McCaughtry, 2014). The theory of physical capital also seems important (Bourdieu, 1978, 2002). Bourdieu (2002) uses the concept of physical capital to show that people take pleasure in positive feedback, such as trust and esteem, in that they are recognized and confirmed as valuable and superior. Physical capital is seen as important in power relationships and for the individual’s status and is a form of capital that could give boys recognition in PE (Bourdieu, 1978). Finally, the theoretical concept of being “seen” in PE may also be central for understanding boys’ experience of having PE in classes with mainly girls. Being seen is a theory in which the students experiencing opportunities to display their skills in PE is one of the factors that constitutes their experiences of being seen.

Our theoretical approach is based on understanding adolescence as a period of identity negotiation in which boys and girls position themselves to different discourses to construct and reconstruct their gender, bodily, and other identities (see, e.g., Pattman et al., 2005; Pattman and Bhana, 2010; Coffey, 2016; Pérez and Salgado, 2018). This approach is related to Foucault’s (1997) concept of “technologies of self” – allowing people to maintain a balance between their personal state and the discourse contexts with continuous negotiations (see Garrett, 2004). For instance, Garrett (2004) finds that girls equate bodily thinness with beauty while considering overweight as ugly and weak. For boys, it is often the question of masculinity compared with the other gender or peers (Tucker and Govender, 2017). Oliver and Lalik (2001) use the term “bodily currency” when referring to the perceived ideal body. This currency has varying value related to bodily hierarchies, which seem to give adolescents social status and popularity among their peers (see also Russell, 2004; O’Brien et al., 2008; Johnson et al., 2013).

A literature search reveals that there are many studies looking at students’ experience of PE from a gender perspective. However, research into boys’ experience of PE in classes in which girls are in a clear majority, seems to be missing. Knowing that boys in girl-dominated classes show up to be the group with the highest level of absence from PE classes (Lagestad et al., 2017) points toward the importance of examining their experiences in PE. Furthermore, understanding the gender regime of any setting gives insight into power relations about how specific groups (such as marginalized boys) place and position themselves (Paechter, 2019). Understanding the issues related to gender is also important to be able to point toward the best approaches for PE in such classes. Furthermore, Gerdin and Larsson (2018) point to the importance of further critical examination of boys’ subsequent exercise of power in PE.

Based on the previous discussion, the aim of the study is to examine the following research question: How do boys in classes with a clear majority of girls in high school experience their PE classes? The conceptualization of gender in this study is problematized according to the findings related to how these boys are presenting the male gender in PE classes with a clear majority of girls.

## Materials and Methods

To shed light on boys’ experience of being a minority in PE, a qualitative design was used, in which boys in such classes were interviewed. An approach using in-depth interviews and student perspectives, together with a phenomenological/hermeneutic approach to data analysis, were seen as being the most fruitful. The phenomenological approach focuses on collecting students’ perceptions of being in the minority. Polkinghorne (1983) refers to the concept of perception as trying to understand the meanings related to one’s experiences. The focus is on a person’s relation to the object or phenomenon to be experienced (Laverty, 2003). Hermeneutic orientation in a phenomenological approach makes an attempt to understand the meanings from historical, contextual, or autobiographical perspectives (see Laverty, 2003).

### Participants

Using a stratified selection, the head teachers from eight of the 16 high schools in Nordland County in Norway were contacted with a request to recruit boys who had PE in classes in which 90% or more of the students were girls. It was also a condition that these students were in the second or third year of high school (16- to 18-year-olds) and who, therefore, had a lot of experience of the question. The teachers of these students communicated the inquiry to their classes, and 13 students from five different high schools agreed to be interviewed. Eleven of the respondents were in classes of “health care,” and two respondents were in “environmental management.” By choosing respondents from different schools and classes – none of the respondents had the same PE teacher – experiences from different school cultures were reported. Five of the 13 students were ethnically Norwegian, and the other eight were immigrated from countries, such as Afghanistan, Eritrea, Somalia, and Serbia. The high number of immigrants in the study reflects that immigrant boys are highly overrepresented in Norwegian “health care” classes. The respondents were part of PE classes with 1–3 boys in each class (class size 20–30), including both female and male PE teachers. The research project was approved by the head teachers of the five schools and the Norwegian Centre for Research Data, fulfilling ethical standards for empirical research. The respondents were fully informed about the protocol prior to participating in the study, and a written consent was obtained from all.

### Procedures

After finishing a review of previous research related to boys’ experiences of PE in classes with a clear majority of girls in school, a semi-structured interview guide was developed, and a pilot interview was conducted with a boy who met the requirements for being a respondent. The interview guide formed the starting point for the interviews. The questions were very general in terms of presenting open and no leading questions, which included questions such as the following: Can you tell me about your experiences in physical education? Do you enjoy physical education? How is it to have physical education in classes with mostly girls? Can you tell me about what you experience as positive and negative aspects related to your physical education? At the same time, when it seemed natural, a number of follow-up questions related to the respondents’ experiences of PE, not included in the interview guide, were posed. The interviews elicited many of the experiences the boys had of PE in classes in which girls were in the clear majority. The interviews were conducted in November and December 2018 by a master student supervised by a professor in physical education. The student researcher was trained in interviewing and qualitative analyses. Respondents’ answers during the interviews were recorded by a digital voice recorder (Olympus DS-50). The interviews lasted between 40 and 60 min. The respondents received information about the research project in advance.

### Analysis

The interviews were transcribed and analyzed with QSR NVivo 10 (London). The analyses were based on a case study approach that included all of the participants’ answers on the questions collected during the interviews, in which students’ perceptions of experiences were taken as subjectively true (Armour and Griffiths, 2012). The data are based on subjective constructions that students verbalized when processing their own interpretations and reflections on what has occurred in PE at school. The analyses are based on transcribed answers focusing on meanings as described by Johannessen et al. (2016). Statements are identified according to the theme of experiences related to being a boy in PE when their gender was in a strong minority and then concentrated, condensed, coded, and categorized in units of analysis and then reconstructed within the theoretical framework (Brinkmann and Kvale, 2015). With such an approach, the students’ statements are assigned codes that are classified into categories (Hastie and Glotova, 2012). The data from the interviews are sorted based on these categories to elucidate patterns, similarities, relationships, or differences between the statements.

The analysis and the interpretation also followed hermeneutical principles in that the interpretation process led to an increasingly deeper understanding of the statements in the interviews according to our comprehension of the whole and the parts (Kvale, 1983).

The transcribed text was read through repeatedly, and five categories were formed out of the interpretations of the students’ statements along the way. Two researchers were engaged in the analysis process, and various alternatives for interpretation and perspectives were assessed. This strategy contributed to intersubjective consensus in the analysis and strengthened the credibility of the findings. By means of coding the students’ expressions, five categories were created: to have to consider the girls, the experience of being physically superior, lack of feelings of mastery, little challenge, and passive girls who get what they want. It seemed preferable to present the results in three related main findings; being physically superior, little challenge and feelings of mastery, and easier to show off. These categories were also seen as the main findings of the study.

## Results

### Respondent Profile

All the informants said in the course of their interview that there is something they are missing in their PE, such as more strenuous activities and a higher physical level among classmates. The majority also said that they miss having more boys in class, especially in the PE classes. In speaking of a lack of boys, the informants often came to the matter of being challenged, having more students at the same level as them, or who are interested in training and ball games. They also mentioned a wish to have a different tempo in PE compared with what they experience today. Each of the 13 informants had been involved in organized sport before and all had participated in team sports. Twelve of the 13 reported being physically active, either in their own fitness regime or in organized sport. All the informants had always had, and still have, a good relationship with PE as a subject to like and look forward to. All reported a good classroom environment, in which they thrive. The informants reported that they always have someone to be with – in both the classroom and PE. The boys feel included and safe at school. The informants liked the classes they are in, and several say that they were clear when they chose the stream they are in when it comes to the number of girls who would choose the same subjects. Some said that it is easier to be social with other boys in the class than with girls. They explained this by saying that they have more common interests and more to talk about.

### Being Physically Superior

All the respondents reported that they miss having more boys in the PE classes, especially pointing toward being physically superior and to have to consider the girls in the PE classes. The boys pointed out that, in relation to the girls, they were taller, quicker, and stronger as well as having better endurance in most cases. The respondents also found themselves to be tougher and more intense in the PE activities compared with the girls. The boys attributed this to genetic differences between women and men, saying that this characterizes the differences they felt, especially in PE. Most of them expressed a wish for – or lack of – more boys in the class, wanting more physical challenges in the lessons. Dan stressed the importance of being a boy in activities when height is an advantage: “We are taller. We can take the ball from over their heads, and we are closer to the basket in, for example, basketball. I notice pretty big differences in large parts of gym.”

Even though the boys reported that they were physically superior to the girls in the PE lessons, a couple of the boys pointed out that, nonetheless, it was possible for the girls to be in better shape than them. Tim said, “It certainly can be that I run fastest, but if we are running a long way, then there are girls in the class who will get away from me.” It is also worth highlighting that each of the 13 respondents had been involved in organized sport before and all had participated in team sports. Twelve of the 13 reported being physically active, in either their own fitness regime or organized sport. Furthermore, all the respondents had always had, and still have, a good relationship with PE as their favorite subject to like and look forward to.

A finding related to the experience of being physically superior was that all 13 respondents reported that they must consider the girls who participated in the PE lessons. To have to consider the girls was described by the boys as several different strategies, such as needing to be careful or acting with less intensity, less power, a slower tempo, and less body contact. In this way, the respondents felt, they must routinely set themselves at a lower skill level than that at which they actually were. Being careful and having to limit oneself meant that that the lessons were more boring and less exciting than PE lessons before. The respondents said that they thought that the instruction was set up so that everyone could cope with the exercises and take part but that the consequence of this was that it became too easy and less challenging for the boys. In this respect, ball games were particularly mentioned. The need to consider came, the boys thought, from this accommodation.

It became clear from the boys’ accounts that having to consider the girls was something the respondents found to be negative because this was experienced as a limitation to their own physical development. Concurrently, the respondents found that giving consideration to the girls in PE classes was necessary. Some of the boys also said that their teacher had been positive and encouraging that the boys show consideration to the girls during PE. An example that makes clear how the boys considered the girls so as not to injure them is Kim’s statement:

I obviously cannot shoulder press them. I do not want to think like that, but it is natural to do so. And so, of course, one becomes more cautious. Obviously do not want to injure anyone. The PE teacher says the same. If he sees that I’m not being considerate, then I’m in bother. I take care when we play ball games also, whether it’s handball, football or basketball. I think about that I cannot throw or center as hard as I would in training, for example.

Tom also indicated that the boys considered the girls and that made the PE less exciting: “It makes us take them into consideration. We hold back a bit, are more cautious. There is not necessarily anything wrong with that, but it makes things a bit boring when we have to do it often.” Jon points to the way in which being considerate negatively affects boys’ performance:

If there were lots of boys and two girls, the girls would do better in gym. But now, when it’s the opposite, I think that we boys maybe become, in fact, worse in gym because we have to be so careful. We must show consideration and we do not want to do any harm, but if it had been the other way around, then perhaps the girls would have had to toughen themselves up a bit to dare to do a bit more. I think that all the girls would have benefitted from that.

Only one respondent, Tim, demonstrates some nuances and tensions in the data related to boys considering the girls, pointing out that the girls in his class were not like other girls; they were “highly motivated to run and to hit the ball with full speed” and “not afraid for the ball or for PE. The girls in my class are really tough,” he claimed – a statement that was in contradiction to the stories of the other respondents.

### Little Challenge and Mastery

To experience having little challenge while together with passive girls who get what they want in PE lessons was another main finding of the analysis. According to the respondents, this negatively affected their feelings of mastery. This was clearly highlighted by all the respondents except Tim in relation to the experience of challenge. Several of the respondents contended that democracy in such a class became unfair when they mostly ended up doing what the girls wanted to do in the cases when the students could choose activities. Julian claimed that “when it is the majority that decides, so we have to go with that because it’s democracy. Of course, we also get heard, but there are more girls in the class, so we mostly do what they want to do.” Ian explains that “much of it is laid down by the teachers and decided following this plan. But often it is democracy that decides. Does not take much to be voted down in my class then.”

A surprising finding was that all the boys said that, when they should work in pairs, they invariably chose another of the boys even though they said that it was no problem to be in a pair with one of the girls. When asked why they did this, they answered that it seemed natural to them and that they felt better able to push each other when they were together in a pair. Some of the respondents added that they would have had more benefit socially had there been more boys in the class.

A finding related to the experience of little challenge, was that the respondents felt that PE was “too easy,” that the lessons offered them insufficient challenge, and that it was seldom that they had a feeling of there being something they had not mastered or with which they struggled. This was because of the low standard set by most of the girls. Many reported that had there been more boys in the class, they could have challenged each other and pushed each other more than was the case, such as Dan:

If I could decide, I would have more boys in the class. I do not want to tread on anybody’s toes but for someone like me who is so keen on sport I think that it would do no harm to have more boys in the class … I think that I personally would have got more out of it, both with regard to the social life of the school and the physical and mental in PE. There would be more to measure oneself against during gym, more to be challenged together with. It’s easier to push yourself when there are others at the same level.

Two of those who are not ethnically Norwegian said that they have feelings of mastery when they are presented with new activities they have not encountered before and succeed in them. When asked if he experienced these feelings of mastery less often in PE now than in previous PE classes (with equal gender balance), Ian answers,

Yes, absolutely. This is just because the level is lower now, compared to what it could have been. And how it would have been had we been more boys. The teacher organizes things in line with the level in the class. We do not shoot with the left foot because we have learnt to shoot with the right, to put it that way. It’s not exactly advanced gym we have. When we have ball games there’s no hocus pocus. The standard is not high enough to be able to take further steps and develop oneself. I felt like we are stagnating.

Again, Tim clearly demonstrated nuances and tensions in the data, pointing out that it was no problem for him experiencing mastery in PE most of the time. Also, Kim highlighted that not every girl was the same and that there were big differences within the group:

Some do try, but others do not give a damn. Some almost never have gym, some come without their gym clothes, some come with excuses for why they cannot take part and so on. Some do have their gym clothes and do try, but there is a huge difference within the class.

In general, all the respondents except Tim pointed to their female classmates as relatively passive during the PE lessons. The boys made the point that many of the girls had little competitive instinct, which the boys saw as a negative, such as Eric:

There are lots here in the class who do not take part in the lessons and this creates a bad atmosphere for the rest of us. It’s because, like, a bit of an infection if there are some who are not with us in gym, so that it becomes OK for others not to be either.

Several of the respondents experienced that there was a lot of giggling among the girls if someone did not manage to do something, combined with a lot of complaining and little effort or interest among the girls if an activity was thought to be difficult or demanding. The respondents emphasized that, if more girls in the class had been interested or engaged in sport (in the way that they themselves were), then perhaps there would have been more interest and effort among the girls. Dan, who appears to be interested in sport and in his own development, explained how it was when he came into a PE class in which there were almost only girls: “Before [in middle school], there was a bit more sportiness among everyone in the class, but now there are only a couple who are engaged in sport and keep fit. There’s a lot of complaining and not much interest in doing what we are asked to do.” When asked what he thought about this, Dan answered, “I think it’s hopeless from my point of view, who wants to be better. But they cope with the training they get, and I get to be involved with the activity even though I do not get the development I should have had.”

That the girls get to choose activities in PE was another finding. “The teacher decides that the majority decides” the boys reported – something that seems unfortunate for the boys who are in the minority. Jon claimed, “We have mostly girls, and so it is often them who form the majority who decide and so it what the girls want to do. I would like it if we could decide as often as the girls, but that is not how it is.” Kim also experiences that it is most often the girls who get to decide when the teacher gives the possibility for participatory decision making:

The first time we had him, he asked us what we liked. He has taken on board something of that. We have to be part of decision making a bit. More so the girls though. There are, of course, more of them than us. We have just said what we like to do, but I think that the girls have got their way much more.

On the few occasions that the boys were “so lucky” as to be able to choose the activities or to have more challenging training, they find that many of the girls contribute poorly and that there is a bad atmosphere. For this reason, many of the respondents said that there was nothing to be said about not being included in decision making because it did not turn out as they had hoped in any case. Several also said that a lot of time was spent on “going through” and explaining, leaving little time to take the exercise further to the next level. They experienced that they are not able to take further steps in the physical training, which they see as negative. They said that this had also occurred to a lesser extent in classes in which there were more boys. In spite of their experiences, the respondents reported to enjoy PE in general.

### Easier to Show Off

Although the first two main findings can be seen as being experiences of the negative side of being a boy in a PE class in which there are mostly girls, the third main finding describes a positive side of being a boy in such a class: that it was easier to show off in front of the PE teacher. For some, like Julian, this was something they had never experienced before, having been in classes where other boys had dominated PE:

It was more difficult to be good at something at middle school – those who were good at football were apt to be good at all the other things too. I enjoy gym at both schools, but it’s easier to do stuff, be involved and show yourself off in the gym lessons here than it was before.

The respondents are clear that they receive more recognition from the PE teacher now than in middle school in more balanced PE classes. In connection with receiving recognition from the teacher, the respondents mentioned receiving feedback as well as being noticed by the teacher. Another positive thing was being a resource or helper for the teacher and the teacher showing that he valued this. This is exemplified by Dan: “I think it’s easier now to be seen in gym than before. I’m good at gym, and I get more of the teacher’s attention now, than when there were more who were equally good as me.”

Ian also thinks that he is more seen by the teacher and speaks about how he can be used as a helper: “It’s easier for the teacher to see what I do for the others, something for which I have had good feedback. I feel like I am a resource for the teacher, and that I can be used to instruct. Contribute to other’s being better.” With a smile on their face, some said that they are also pleased that the girls see them as strong and skillful. Tom is pleased to be one of those who is best at gym, and sees that this is because there are few boys in his PE class:

The girls see us as able and quick, but if there had been more boys, then for sure some of them would have been better than me. When we are so few boys, then I get to be one of the best, but, if there were more of us, then I would not. I think this is good – that I get to see myself as the best, and that the others also get to see that I am the best.

## Discussion

Analysis of the data point to three main findings. Two of them can be characterized as being negative in nature. The analyses showed that of the 13 respondents, only one respondent, Tim, demonstrates some nuances and tensions in the data as highlighted in the results. The findings are discussed separately before discussing the perspective of producing a traditional male gender in PE.

### Being Physically Superior

The analyses show that the boys in general described themselves as taller, quicker, stronger, being in better physical shape, and tougher than the girls with whom they had PE – in other words experiencing themselves as physically superior in PE.

Biological gender is important because weight and size are linked to endurance, strength, and mobility, which contribute to the basis of physical capability (Hansen, 2005). Researchers also point out that boys’ physical superiority and size gives them the possibility of dominating in PE (Evans, 1989; Lagestad, 2017). The respondents’ stories clearly support these studies. On the other hand, PE teachers report that boys are likely to overestimate their physical ability in PE and are unwilling to include girls in PE (Barker, 2019).

It is appropriate to note that physical capabilities are central to the accomplishment of activities in PE (Klomsten, 2013). PE is a subject in which the students are physically active through bodily interaction in, for example, competitions, games, and cooperative exercises. PE is, therefore, one of the arenas in which students consistently see the sexes in relation to one another and compare themselves to others through, among other things, physical capability. Here, it seems natural to draw on the concept of physical capital (Bourdieu, 1978) and body as a currency (Oliver and Lalik, 2001) – both concepts pointing out that boys have more physical capital than girls and that this is the basis for their dominance or status in PE classes. Research shows that Norwegian PE is dominated by sport activities, especially ball games (Standal et al., 2020). One study also concludes that PE in Norway seems to favor students who are involved in sport activities (Säfvenbom et al., 2014). Our results support these findings, indicating that there is a need for a new strategy in Norwegian PE. This is in line with Gerdin and Larsson (2018), who conclude that the focus on certain discursively constructed bodily practices continues to restrict diversity in PE and that traditional gender patterns are reproduced through a selection of particular sports and physical activities in which all the students are expected to participate. We suggest that activities other than sport-based activities must be included in PE – activities that do not require strength and other athletic skills, leading to feelings of hegemonic masculinity. Research reveals that altered pedagogical practices and relationships result in increasing student engagement in PE (Garrett and Wrench, 2018). A new approach of teaching that is more student-centered with more focus on social interactions during PE classes and in which students’ sex is not as important as in traditional, teacher- and sport-centered PE classes, seems more appropriate. A study that indicates PE teachers are engaged in teaching practices that reinforce gender stereotypes through gender segregation (Valley and Graber, 2017) supports this conclusion. Studies points to how involving students in PE lessons is very important for students’ experiences of meaningfulness, achievement, and well-being in PE (Garrett and Wrench, 2018). Furthermore, our results may also point to the importance of the Teaching Games for Understanding approach (O’Leary, 2016). The Teaching Games for Understanding approach points to children’s inherent desire to play and highlights that every student is important and must be involved. This approach attempts to move the emphasis from an individual skills performance to a team-based, student-centered approach.

To have to consider the girls during PE is about being more careful, acting with less intensity and power, and with a slower tempo and less body contact. In this way, the respondents felt like they were routinely setting themselves at a lower skill level than that at which they felt they actually were. In light of flow-zone theory (Csikszentmihalyi, 1975), this negative experience of having to consider the girls can lead to one experiencing things as boring, and according to flow-zone theory, this is a poor basis for growth. From a critical point of view, the PE sessions could be perceived as “low level” also by the girls. However, it is difficult to know without further studies of girls. In this study, we were only focusing on the boys’ experiences.

### Little Challenge and Mastery

Our findings show that boys in general find themselves very little challenged and that the respondents experience much of their PE as boring and demotivating. They did not experience mastery because they thought the lessons were too easy and did not give much challenge. As pointed out in the earlier discussion of flow theory, many respondents found that there is a flow zone, which is a fantastic state to be in; a state that one would like to experience again (Csikszentmihalyi, 2008). People in the flow zone experience a balance between the challenge with which they are confronted and the competence they have in this area. When one masters something, an inner motivation is created, and one wants to achieve more such things. When there is an equivalence between the challenges in the PE lessons and the boys’ ability, there are feelings of mastery, giving them energy and desire in the lessons. In the absence of mastery, boys come out of that flow zone, which, according to Csikszentmihalyi, quickly leads to a state characterized by boredom and frustration. This can only be seen as highly unsatisfactory given that the aim of the subject is to inspire a life-long pleasure in movement (Utdanningsdirektoratet, 2019).

To be inspired to physical movement, the study plan for PE acknowledges the need to experience joy and mastery (Utdanningsdirektoratetet, 2011, 2015a), and this stands in agreement with the boys’ experience of insufficient opportunity for mastery in PE as problematic. This is because research has found that participation in movement activities over time requires a certain degree of pleasure (Dismore and Bailey, 2011). The study shows that boys with a high level of physical conditioning have the expectation that their PE lesson is a kind of training session and like such an approach (Bjerke et al., 2016). That the respondents seem to be in good physical shape and nonetheless found little in their PE classes to be physically demanding can help us to understand their poverty of feelings of mastery. Earlier research finds that having too many girls in a class can be negative in that many of them do not do their best in the activities (Black et al., 2013). Boys can also see girls as a limiting factor in performance and effort in PE, which can lead to them giving less of themselves in the activities (Klomsten, 2013) than they most probably would have done had the composition of the class been different. It is problematic that our respondents have so little experience of being heard and that those thought of by the boys as “passive girls” get to decide the intensity of the activities.

### Easier to Show Off

We remarked earlier that boys who found themselves feeling as physically superior experienced that negatively while, at the same time, several of them also said that they liked to be the best in the class as well as liked the attention coming of being physically superior. This finding can be explained by the theory of being seen and the importance of opportunities for students to display their skills in PE (Lagestad et al., 2020). The theory of being seen in PE includes several aspects, but the opportunities for students to display their skills in PE seems especially important here. The finding can also be explained by Bourdieu (2002) and his concept of physical capital, in which boys get positive feedback from their PE teacher because they are recognized and confirmed as valuable and superior. The boys indicated that it was easier to show off in PE in classes when there was a majority of girls. This result marks itself as the only one of the main findings in which the respondents name a positive aspect of having PE in a class dominated by girls. The interviews indicated that the boys felt that they were seen differently by the PE teacher than when they were in more gender-balanced classes. That the respondents in this study experienced being seen as positive is in agreement with Lagestad et al. (2020). In Lagestad (2017), a girl who was the only girl in her high school class reported that she was seen by the PE teacher but that she experienced this as negative. Another study suggested that having a high activity level, such as our respondents had, was a good means of being noticed by the PE teacher (Lagestad et al., 2020). To this extent, this supports the findings of this study.

### Producing a Traditional Male Gender in PE

Paechter (2019) highlights that gender research allows us to examine how dominant narratives about what it is to be a minority boy in PE affect how different forms of behavior are constructed, which seems to be the case according to the respondents in the present study. According to the 13 boys’ experiences in PE classes with a majority of girls, they seem to have done “identity work” within gender in PE that is closely related to “doing boy” in a traditional manner (Azzarito and Katzew, 2010). Also, Tischler and McCaughtry (2011) highlight PE as an arena for “doing boy right,” pointing toward masculinity being viewed as strong, powerful, fit, and fast as a key feature of the PE environment. The findings can be related to a story of how the respondents present a traditional male gender in PE through their mastery, strength, and ambition to compete.

In her research, Hill (2015b) also shows how strength and body were the key elements in the PE among the boys she interviewed, giving them status in front of others. Even if there are some nuances, all boys except one very clearly point to girls in general being at a lower level, according to both physical skills and motivation to work hard in PE or taking PE “seriously.” The narratives of the boys point clearly toward hegemonic masculinity (Connell and Messerschmidt, 2005; Tucker and Govender, 2017). As pointed out in the introduction, hegemonic masculinity is a relational concept that is produced as the dominant concept that legitimates inequitable hierarchical relations between men and women. The universal characteristics of hegemonic masculinities in PE are related to strength, speed, muscularity, and competitiveness – characteristics the respondents highlighted as important factors in their narratives about themselves. Our findings support many other PE studies that show how embodying characteristics, such as strength, speed, and desire to compete, are being produced as dominant (Davison, 2000; Drummond, 2003; Tischler and McCaughtry, 2011, 2014; Hill, 2015b). The findings also support Connell and Messerschmidt (2005), arguing that bodies play a major role in boys experiences in a social context and that adolescents’ experiences of themselves are shaped by their bodies. In this way, bodily performance serves as a key marker of how to perform boy right (Tischler and McCaughtry, 2014).

By doing traditional sport activities in PE together with girls, the respondents achieved and experienced “the successful body” as highlighted by Drummond (2003) – a body that is highly skilled in sport and physical activity and being outstanding at sport is “a taken-for-granted concept.” Subsequently, Tischler and McCaughtry (2014) found that, in the context of sport-based PE, which privileged boys with athletic traits, such as strength and speed, masculinity hierarchies, produced, putting the sporty boys on the top. In this way, the sport-based PE legitimated hegemonic masculinities. As pointed out by Tischler and McCaughtry (2011), when teachers emphasized elite performance and competition, they inevitably privileged boys who enjoy competition and whose skills align with their teaching practices. Studies show that PE teachers report having limited training related to gender issues in PE and are largely unaware of the link between gender and inclusion (Lleixàa and Nieva, 2020).

Klomsten (2013) emphasizes that boys often make greater efforts in activities because they measure their physical strength against each other. This can lead to activities undertaken at speed and with power, something which, on the other hand, is seldom the case in girl-dominated classes in which boys are in the majority. This was confirmed by our respondents and is something that most of them link to having to be considerate to the girls.

In relation to creating a traditional male gender role in PE, the fact that eight of the 13 respondents had immigrated from countries in which women are discriminated against, may have an impact on their understanding of gender. In a study of PE teachers’ stories about PE, the teachers reported that boys from other cultures saw themselves as superior to girls (Barker, 2019). On the other hand, the stories the ethnic Norwegians and the immigrants told did not differ.

It can be argued that our findings may help reinforce essentialists’ notions of boys (and girls) and what they are or should be like. There is a wealth of research on boys’ and girls’ experiences in PE over the last decades that have challenged the essentialist notions and provide more nuanced accounts of gendered experiences in PE. We agree with Paechter (2019), who argues that there are frequently groups of boys that do not aspire to the local hegemonic masculinity, but in our study, those boys were not present. However, our sample of respondents is very limited, and as highlighted in the methods section, in this study, we have taken respondents’ interview stories as subjectively true (Armour and Griffiths, 2012). It could be an essentialist notion pointing to boys as a homogeneous group when previous research in this field has critiqued this view for decades. However, our findings indicate that, in relation to the respondents’ stories, all of the respondents seem to be in broad agreement in their experiences of being a boy in a girl-dominated PE class. We argue that being a boy in PE classes doing traditional sport activities with mainly girls may lead these boys to construct their masculinity into the form of a hegemonic masculinity. Tischler and McCaughtry (2011) point out that some boys are marginalized in PE settings when hegemonic masculinity is present, making boys feel that PE is not for them – an experience that should be worrying PE teachers all over the word. Finally, it is worth highlighting that all of the respondents were physically active in their spare time, and boys that do not participate in such activities might have perceived PE differently. To what extent the findings regarding boys attending PE classes with a majority of girls is representative of experiences regarding boys attending gender-balanced PE classes is difficult to determine. We suggest that being a boy in PE classes doing traditional sport activities may lead many boys to construct their masculinity into the form of a hegemonic masculinity also in gender-balanced PE classes, but this construction is probably less obvious or clear.

### Strengths and Limitations of the Study

In total, 13 students from five different high schools were interviewed for this study. In a qualitative study using in-depth interviews, it is a high number, giving insight into experiences from different schools and different PE teachers. That all data seem to confirm the same main findings strengthens the credibility and reliability of the study. One must be careful in generalizing findings, though. This might require quantitative studies, including statistical analyses. Nonetheless, the findings have a credible general validity on the basis that all 13 respondents seem to be in broad agreement in their experiences of being a boy in a girl-dominated PE class.

A weakness of the study is that it includes only the perspectives of students. With our approach, we did not ask the teachers about their experiences or perceptions. However, the research question we set for ourselves sought data only on this perspective. Furthermore, a lack of random selection of the participants is a limitation of the study. From this perspective, the respondents may not represent boys in general. Most of the respondents were physically active, had a good relationship with PE, and many were immigrant boys – a fact that probably affected their experiences of PE.

## Conclusion

This is the first study to examine boys’ experiences of PE in classes in which there is a significant majority of girls. Three main findings were revealed from the analyses, namely (1) being physically superior and having to consider the girls, (2) little challenge and feelings of mastery together with passive girls who get what they want, and (3) easier to show off as a boy. We argue that our findings show the minority boys produce a traditional male gender in PE. This result was discussed in relation to achieving a hegemonic masculinity. In the light of the finding, we agree with conclusion of Drummond (2003), pointing out that the need to challenge the hegemonic nature of sports and masculinized sport must be a goal for all physical educators. Knowing that PE teachers report having limited training related to the link between gender issues in PE and inclusion (Lleixàa and Nieva, 2020) is of great importance. We suggest a new approach to teaching that is more student centered. A strategy could be to include activities other than sport-based activities in PE, activities that do not require strength and other athletic skills leading to feelings of hegemonic masculinity. Furthermore, a larger focus on social interactions during PE classes, in which students’ sex is not as important as in traditional teacher- and sport-centered PE classes, may be part of the strategy. Our results indicate that it is important that physical educators working with students have a practical understanding of the critical theories associated with hegemonic masculinities and what the students’ bodies mean to them in the context of sport and physical activities as highlighted by Drummond (2003). We hope our findings enhance the development of PE teachers’ pedagogical understanding and skills. Further research should include teachers’ perspectives or include additional participants by means of a quantitative study design to be able to generalize from boys’ experiences of being a minority in the PE lessons. Finally, intervention studies, including new approaches of teaching with more focus on social interactions and more student-centered PE in which students’ sex is not as important as in traditional, teacher- and sport-centered PE, are important.

## Data Availability Statement

The raw data supporting the conclusions of this article will be made available by the authors, without undue reservation.

## Ethics Statement

The studies involving human participants were reviewed and approved by the Norwegian Centre for Research Data. Written informed consent to participate in this study was provided by the participants. Written informed consent was obtained from the individual(s) for the publication of any potentially identifiable images or data included in this article.

## Author Contributions

PL has contributed on design and methods, writing the introduction, materials and methods, discussion, and conclusion, and furthermore, a critical review of all the text during several numbers of the article and rewriting of the text. ER has contributed on writing the introduction and discussion, and a critical review of the text and rewriting of the text. TB has contributed on design and methods, writing the introduction, materials and methods, discussion, and conclusion, and a critical review of the finale manuscript. All authors contributed to the article and approved the submitted version.

### Conflict of Interest

The authors declare that the research was conducted in the absence of any commercial or financial relationships that could be construed as a potential conflict of interest.
